# Validation of a Farsi version of the Eating Pathology Symptoms Inventory (F-EPSI) among Iranian adolescents

**DOI:** 10.1007/s40519-023-01561-4

**Published:** 2023-03-27

**Authors:** Reza N. Sahlan, Jessica F. Saunders, Ellen E. Fitzsimmons-Craft

**Affiliations:** 1 Department of Clinical Psychology, Iran University of Medical Sciences, Tehran, Iran; 2 Psychology Convening Group, Ramapo College of New Jersey, Mahwah, USA; 3 Department of Psychiatry, Washington University School of Medicine, Washington University in St. Louis, Mailstop 8134‐29‐2100, 660 South Euclid Avenue, St. Louis, MO 63110 USA

**Keywords:** Eating Pathology Symptoms Inventory, F-EPSI, Assessment, Iran

## Abstract

**Purpose:**

Limited research has validated eating pathology assessments in Iranian adolescent boys and girls. In particular, the measures that have been validated do not capture both boys’ and girls’ eating behaviors in adolescence. The purpose of the current study was to validate a Farsi version of the Eating Pathology Symptoms Inventory (F-EPSI) for use in Iranian adolescents.

**Methods:**

Participants (*N* = 913; 85.3% girls) were adolescents who completed a battery of questionnaires including the F-EPSI. In addition, F-EPSI data for Iranian adolescents were compared with those of previously published data of adult Iranian college students.

**Results:**

Confirmatory Factor Analysis (CFA) indicated that the F-EPSI had an acceptable fit to the data and supported the eight-factor model. The scale was invariant by gender, weight status, eating disorder, and age groups. Boys reported higher scores than girls on the Excessive Exercise, Muscle Building, Body Dissatisfaction, and Binge Eating subscales. Adolescents with higher weight and eating disorder symptoms endorsed higher scores on the F-EPSI subscales. Older adolescents and adults reported higher scores than younger adolescents and adolescents, respectively. Adolescents had higher scores than adults on Restricting and Excessive Exercise subscales. The F-EPSI demonstrated good convergent validity through correlations with other eating pathology symptoms. The F-EPSI subscales were associated with depression and body mass index (zBMI) in expected directions that indicate criterion validity of the scale.

**Conclusions:**

Findings suggest that the F-EPSI is a reliable and valid measure in Iranian non-clinical adolescents. The F-EPSI will enable researchers to examine a broad array of eating pathology symptoms in adolescents for whom Farsi is their official language.

**Level of evidence:**

Level V; Cross-sectional descriptive study.

## Introduction

Studies reported that eating pathology is common among Iranian adolescents, with potentially important gender differences [1, 2]. For example, 20% of adolescents reported recurrent (≥ 4 times during the past 28 days) binge eating and 4–7% reported current purging behaviors (self-induced vomiting, laxative misuse) [2]. In addition, levels of dietary restriction and cognitive features of eating pathology^1^ are higher in adolescent girls than boys [1, 2]. However, binge eating, purging behaviors, and excessive exercise levels have been found to be comparable across boys and girls [1], as has eating pathology-related impairment (i.e., 17.4%; [3]). Therefore, it is important to have measures that are psychometrically valid and equivalent across genders to improve our understanding of eating pathology in adolescents.

To our knowledge, there are three Farsi scales assessing eating pathology among Iranian preadolescents and adolescents: the Eating Disorder Examination Questionnaire (EDE-Q; [1, 4]), Eating Attitudes Test-26 (EAT-26; [5]), and Children EAT-20 (ChEAT-2; [6]). However, there are some limitations of existing scales in Iran. First, the original factor structure of the EDE-Q has not yet been examined in Iran. Second, the EDE-Q, EAT-26, and ChEAT-20 assess eating pathology symptoms (e.g., thinness/dietary restraint) that are common in girls and fail to assess those that are more common in boys (e.g., muscularity/exercise; [7]). Third, the EDE-Q, EAT-26, and ChEAT-20 assess behavioral features of eating pathology using single items. Importantly, with respect to the EDE-Q, behavioral features of eating pathology (i.e., binge eating, purging, excessive exercise) are assessed using count variables that do not allow researchers to examine their factor structure [8, 9]. Thus, authors have only been able to examine the factor structure of the four EDE-Q subscales [8]. Therefore, these findings indicate the need for measures that assess a broader spectrum of eating pathology to be administered in Iranian adolescents.

The Eating Pathology Symptoms Inventory (EPSI) was validated among adolescent boys and girls (age 13 through 17) in the United States (US) to address the above-mentioned limitations by capturing multiple dimensions of eating pathology cognitions and behaviors, including body dissatisfaction, binge eating, cognitive restraint, purging, restricting, and excessive exercise [10]. The EPSI has demonstrated a robust six-factor structure among adolescents in the US [10]. In addition, there have been some gender differences identified among adolescents [10]; however, measurement invariance across genders was not established. Despite this limitation, the scale was invariant across age groups (i.e., younger adolescents vs. older adolescents and adolescents vs. adults) [10]. There have been some age group (i.e., adolescents vs. adults) differences on the EPSI subscales. The EPSI was invariant across younger vs. older adolescents; however, mean level differences were not examined across these two age groups. Mond et al. [11] reported that older adolescents reported higher eating pathology symptoms (assessed through the EDE-Q; (12) than younger adolescents. Relatedly, Sahlan et al. [6] reported that age had a positive association with eating pathology in preadolescents. Similarly, Hatami et al. [13] found that increasing body dissatisfaction was associated with increasing age and weight status in Iranian adolescents; however, the measurement invariance of this scale was not established by either age or weight status. To our knowledge, there are three studies in which eating pathology assessments such as Loss of Control Over Eating Scale (LOCES; [14]), Clinical Impairment Assessment (CIA; [3]), and ChEAT-20 [6] were invariant by weight status and age among Iranian preadolescents and adolescents.

Regarding the importance of the EPSI within an Iranian context, Richson et al.’ [10] study had two main limitations. Given that two EPSI subscales, muscle building and negative attitudes toward obesity, were omitted by the creators of the recovery record mobile phone application, Richson et al. [10] did not include those subscales upon assessing adolescents’ eating pathology. However, muscle building and negative attitudes toward obesity are of importance in adolescents. Ricciardelli and McCabe [15] reported that muscularity is of importance while examining eating pathology in adolescents. Relatedly, a few studies demonstrated that muscularity was associated with eating pathology symptoms in adolescent boys and girls and boys endorsed higher muscularity than girls [16, 17]. In line with Western adolescents, it has been found that body-related physical functioning was one of the main physical components of perceived body image among adolescents in Iran [18]. For example, body change behavior, expressed through drive for muscularity, was common among adolescents (41.1%) in Iran [19], thus demonstrating the effects of body image concerns on muscularity among this population [20]. In addition, Bahrami et al. [21] reported that 5–10% of adolescents in Iran consumed dietary supplements meant to reduce weight. These findings may indicate the importance of muscularity and related components that could contribute to eating pathology which is common among this population in Iran. In accordance with muscularity, 43% of Western adolescents found that it is disgusting when a fat person wears a bathing suit at the beach, which indicates the importance of negative attitudes toward obesity in adolescence [22]. The negative attitudes toward obesity were not related to body mass index (BMI) in adolescents and adolescent boys reported higher negative attitudes toward obesity than adolescent girls [22]. In a qualitative study, Kavehfarsani et al. [23] found that adolescent boys and girls in Iran had negative attitudes (i.e., ridicule, etc.) toward individuals with higher weight. Altogether, it may be crucial to examine both muscularity and negative attitudes toward obesity subscales, along with other subscales upon testing the EPSI’s factor structure in adolescents.

To our knowledge, there is only one study in which the EPSI in Farsi version (F-EPSI) was administered to Iranian college men and women [24]. The results indicate that the EPSI subscales demonstrate a robust eight-factor structure in college students [24]. In addition, the EPSI subscales were correlated with loss of control over eating, clinical impairment, and BMI, in support of the EPSI’s convergent validity in this population [24]. However, these findings are not generalizable to adolescents. Notably, in spite of the prevalence of eating pathology among adolescents and adults [2], it is unclear whether adults vs. adolescents and individuals with and without eating disorder symptoms would conceptualize eating pathology symptoms in the same way. Moreover, two studies in Iranian adolescents [14] and college students [24] reported that individuals’ weight status related to eating pathology symptoms and individuals with higher weight reported higher loss of control eating [14]. However, with respect to other eating pathology symptoms, little is known within an Iranian literature. There is one study in which Forbush et al. [25] found that the EPSI was invariant by weight status among the US adults. Taken together, examining the measurement invariance of the EPSI subscales by age (i.e., adolescents vs. adults), BMI, and eating disorder groups is warranted. Notably, there have been some differences across groups while assessing eating pathology. However, it is imperative to know whether the scales perform similarly across groups and that observed group differences are due to *true discrepancies* and not *measurement error* [26]. More precisely, having a consistent measure of eating pathology across the lifespan would better characterize developmental changes in eating pathology in both research and clinical settings. Therefore, measurement invariance would be warranted to respond to these gaps. Given the strong psychometric properties of the EPSI and its potential to capture diverse eating pathology symptoms in both boys and girls, it would be beneficial to use this assessment to examine eating pathology in Iranian adolescents. The purpose of the current study was to examine the factor structure, measurement invariance, and other psychometric properties (i.e., convergent validity; internal consistency) of the F-EPSI. We postulated the original eight-factor structure of the EPSI would replicate in the Farsi version ([27]; **H1**) among adolescents. Consistent with previous research among the US adolescents [10, 16], adults [25], and Iranian college samples [24], we expected that the F-EPSI factor structure would be invariant across genders, weight status, and age (i.e., younger vs. older adolescents; adolescents vs. adults) groups (**H2**). Furthermore, we hypothesized that the EPSI subscales would demonstrate strong reliability (i.e., Cronbach’s alpha, McDonald’s ω; ≥ .70) in adolescents ([10]; **H3**). If the scale was invariant across groups, in addition to our primary objective, we had three secondary objectives related to the validity of the F-EPSI. We first posited that girls would report higher scores on body dissatisfaction and cognitive restraint, while boys would endorse higher restricting and muscularity [10, 16]. Second, individuals with higher weight would report higher scores on the F-EPSI subscales, except for restricting and muscle building [24]. Third, adolescents would endorse higher scores on body dissatisfaction, restricting, cognitive restraint, purging, and excessive exercise compared to adults, while adults would report higher scores on binge eating compared to adolescents ([10]; **H4**). In accordance with the EPSI’s data among US [25, 27], Chinese [28], and Iranian [24] adults, we also expected that most of the F-EPSI subscales would have a strong, positive correlation (*r* ≥ .40) with a global score of the EDE-Q, binge eating, purging, over-exercise, depression, and BMI, while the restricting and muscle building subscales would demonstrate weak, negative correlations with BMI (*r* ≥ − .10 [29]; **H5**). Finally, we explored measurement invariance of the F-EPSI subscales among adolescents with and without clinically significant eating disorder symptoms. If the scale is invariant across groups, adolescents with clinically significant eating disorder symptoms may report higher scores on the F-EPSI subscales. This final analysis was exploratory.

## Methods

### Participants and procedure

Participants (*N* = 913; 85.3% girls) were adolescents who were recruited from three cities with diverse geographic zones and ethnicities (i.e., Tehran _*n* = 288 (31.5%)_ [Capital, Persian], Tabriz _*n* = 308 (33.7%)_ [North-Western, Turkish], and Kurdistan _*n* = 317 (34.7%)_ [West, Kurdish]). The age range of participants was 10–19 years (*M* = 15.07, *SD* = 1.90). Standardized body mass index (zBMI) ranged from − 2.20 to 5.70. Analysis of variance (ANOVA) indicated no significant differences in age (*F* (2910) = 1.11, *p* = .329) or zBMI (*F* (2910) = .09, *p* = .919) by city. Using an online method (i.e., Google forms) advertised in schools’ social media groups (i.e., Shad program,^2^ WhatsApp, Telegram), interested adolescents completed a battery of questionnaires without any financial remuneration from March 2021 to February 2022. All questionnaires were anonymous with no identifying information collected to protect the confidentiality of participants. The school and regional administrators approved the research procedures. Parental consent was obtained prior to participation, and adolescents provided assent. The study was approved by the institutional review board from a large Iranian university.

For the comparisons with Iranian adults, we used Sahlan et al.’s data [24] in which college students (*N* = 765) were recruited from Iran. College students ranged in age from 18 to 28 years (*M* = 21.12, SD = 1.20) and self-reported BMI ranged 15.16–34.95 kg/m^2^ (*M* = 23.40, SD = 3.81; see Sahlan et al. for review; [24]).

### Measures

#### Demographic information

Participants reported their age, gender, height, and weight (used to derive BMI; kg/m^2^; [30] on a study-specific questionnaire. Aligned with the World Health Organization guidelines for children and adolescents aged 5–19 years, we converted BMI to age- and sex-specific zBMI scores.

#### Farsi Eating Pathology Symptoms Inventory (F-EPSI)

We used a Farsi version of the F-EPSI (F-EPSI; [24]) that assesses eight subscales: Body Dissatisfaction, Binge Eating, Cognitive Restraint, Excessive Exercise, Restricting, Purging, Muscle Building, and Negative Attitudes toward Obesity. The items are rated on a five-point scale ranging from 0 (*Never*) to 4 (*Very often*) and higher scores indicate a greater level of eating pathology [10, 25, 27]. The validity and reliability of the F-EPSI has been supported among Iranian college samples [24] for the first time, using a scale that underwent translation and back-translation procedures. In addition, prior to conducting the study, we piloted the F-EPSI with a small group of adolescents (boy *n* = 20, girl* n* = 19) to ensure adolescents could read and comprehend all the items and found that all the items were understandable to adolescents.

#### Farsi-Eating Disorder Examination Questionnaire (F-EDE-Q)

We used a Farsi version of the EDE-Q (F-EDE-Q; [1, 4]) that assesses ED symptoms over the past 28 days. Twenty-two items are rated on a seven-point scale ranging from 0 (*No days*) to 6 (*Every day*). Five items assess the frequency of ED behaviors. Higher scores indicate a greater level of pathology. The validity and reliability of the F-EDE-Q has been supported in Iran [1, 4]. In the current study, we focused on a global score of the F-EDE-Q, binge eating, purging, and over-exercise. In line with a previous study in Iran [31], we summed self-induced vomiting and laxative misuse items to provide one purging frequency index. Cronbach’s α (.93) and McDonald’s ω (.94) were reported for a global score of the F-EDE-Q in the current sample. McDonald’s ω was .85 for purging in the current sample.

#### Farsi-Revised Children Anxiety and Depression Scale (F-RCADS)

We used a Farsi version of the RCADS (F-RCADS; [32]) that assesses anxiety and depressive symptoms. The Depression subscale includes ten items that are rated on a four-point scale ranging from 0 (*Never*) to 3 (*Always*). In the current study, we used the Depression subscale. The validity and reliability of the F-RCADS has been supported in Iran [32]. Higher scores indicate a greater level of depression. Cronbach’s α (.91) and McDonald’s ω (.92) were reported in the current sample.

### Analytic plan

There were no missing data on any of the scales administered. We first conducted confirmatory factor analysis (CFA) to test the original 8-factor structure of the F-EPSI in adolescents (**H1**). Consistent with prior research [25], we employed robust Diagonally Weighted Least Squares (DWLS), given the ordinal nature of the data. The weighted least squares estimator (WLSMV) is a robust version of the DWLS in JASP [33]. As suggested in the literature [34, 35], model fit was assessed using the following indices: root mean square error of approximation (RMSEA; < .08 indicates a good model fit), the comparative fit index (CFI; > .90 indicates an acceptable model fit), the Tucker–Lewis index (TLI; > .90 indicates an acceptable model fit), and the standardized root mean square residual (SRMR; < .08 indicates a good model fit).

We used multi-group confirmatory factor analysis (MGCFA) to test the measurement invariance by gender, zBMI status (i.e., low-to-average weight [zBMI <  + 1SD] vs. higher weight [zBMI >  + 1SD]), eating disorder symptom clinical cutoffs, and age (i.e., younger adolescents [10–15] vs. older adolescents [16–19]; adolescents vs. college samples), to assess whether the scale performed similarly for individuals by gender, weight status, and age groups (**H2**; [36]). With respect to eating disorder groups, we used a cutoff of EDE-Q global score (i.e., ≥ 2.50; [37]) in differentiating adolescents with and without clinically significant eating disorder symptoms. Measurement invariance was assessed at the configural, metric, scalar, and strict levels [38]. Configural invariance implies that the latent F-EPSI variable(s) and the pattern of loadings of the latent variable(s) on indicators are similar across genders (i.e., the unconstrained latent model should fit the data well in both groups). Metric invariance implies that the magnitude of the loadings is similar across genders; this is tested by comparing two nested models consisting of a baseline model and an invariance model. Scalar invariance implies that both the item loadings and item intercepts are similar across genders and is examined using the same nested-model comparison strategy as with metric invariance [29]. Finally, strict invariance implies that factor loadings, intercepts, and residual variances are fixed across groups [39]. Following the recommendations of Cheung and Rensvold [40] and Chen [41], we accepted ΔCFI ≤ .010, ΔTLI ≤ .010, and ΔRMSEA ≤ .015 or ΔSRMR ≤ .010 (.030 for measurement invariance) as evidence of invariance, as chi-square is not a robust indicator in large samples. We used JASP version 0.14 to conduct CFA and measurement invariance.

Cronbach’s alpha and classical McDonald’s ω (i.e., for categorical variables) assessed internal consistencies (**H3**; [42, 43]). The following cutoffs were used to indicate good internal consistency: Cronbach’s α and McDonald’s ω ≥ .70 [43, 44]. We calculated item reliability to provide an estimate of the extent to which an individual item is a reliable indicator of the underlying construct by squaring each standardized factor loading from the CFA.

Upon establishing measurement invariance across groups using MGCFA, independent sample *t* tests (Normality of data) and Mann–Whitney U (Non-normality of data) tests examined mean differences of the F-EPSI subscales scores by gender, weight status (**H4**), eating disorder groups, and age across groups. As proposed by Cohen [29], *d* = 0.20 is interpreted as small effects, *d* = 0.50 as medium effects, and *d* = 0.80 as large effects for both independent sample *t* tests and Mann–Whitney U tests. We used SPSS 25.0 for these analyses.

To establish convergent validity of the F-EPSI (**H5**), Pearson (Normality of data) and Spearman (Non-normality of data)’s correlations examined associations between F-EPSI subscales, eating disorder symptoms, binge eating, and purging. To test criterion validity of the F-EPSI (**H5**), Pearson’s correlations examined associations between F-EPSI subscales, zBMI, and depression. According to Cohen [29], correlation coefficients of .10, .30, and .50 are considered to be small, medium, and large correlations, respectively. We used SPSS 25.0 for these analyses.

## Results

### Confirmatory factor analysis

We tested normality of the F-EPSI items using skewness, and kurtosis values, and found that purging and muscle building subscales were not normal (see Table 1 for more information). In support of **H1**, the original 8-factor structure of the EPSI demonstrated acceptable fit to the data in the overall sample. Standardized factor loadings ranging from .43 to .82 (see Table 1 for more information). Model fit statistics and change in fit values from the multi-group CFAs are included in Table 2.

**Table 1:** Means, standard deviations, skewness, kurtosis, standardized factor loadings, and item reliability of the F-EPSI items

		*M*	SD	Skewness	Kurtosis	Standardized factor loadings	Item reliability
Body dissatisfaction	EPSI-1	1.18	1.13	.74	− .14	.46	.21
	EPSI-12	.86	1.12	1.17	.42	.70	.49
	EPSI-18	1.21	1.26	.76	− .52	.82	.68
	EPSI-23	.72	1.10	1.50	1.36	.50	.25
	EPSI-24	1.10	1.36	.95	− .44	.75	.57
	EPSI-25	1.45	1.44	.56	− 1.04	.80	.65
	EPSI-34	.84	1.23	1.41	.88	.66	.44
Binge eating	EPSI-3	1.11	1.14	.78	− .30	.59	.35
	EPSI-9	.98	1.05	.89	.06	.53	.28
	EPSI-19	.76	1.01	1.29	1.0	.73	.53
	EPSI-28	.72	1.04	1.51	1.60	.64	.41
	EPSI-37	.92	1.05	1.18	.89	.51	.26
	EPSI-39	.40	.82	2.44	6.16	.57	.32
	EPSI-44	.53	.90	1.85	3.07	.66	.43
	EPSI-45	.66	.87	1.26	1.10	.70	.48
Cognitive restraint	EPSI-2	2.02	1.16	− .03	− .72	.60	.36
	EPSI-21	.87	1.04	1.08	.52	.65	.43
	EPSI-40	1.44	1.20	.48	− .66	.76	.58
Purging	EPSI-11	.09	.43	5.62	34.97	.63	.40
	EPSI-13	.26	.69	2.98	8.94	.48	.23
	EPSI-16	.25	.67	3.05	9.69	.58	.33
	EPSI-17	.09	.37	4.57	20.76	.68	.47
	EPSI-27	.06	.30	6.80	58.64	.61	.37
	EPSI42	.05	.30	7.33	64.68	.71	.51
Restricting	EPSI-4	1.34	1.25	.61	− .67	.46	.21
	EPSI-6	1.21	1.16	.69	− .42	.54	.29
	EPSI-10	2.04	1.05	− .06	− .51	.48	.23
	EPSI-33	1.60	1.11	.31	− .64	.70	.49
	EPSI-36	1.32	1.22	.61	− .62	.59	.35
	EPSI-43	1.12	1.09	.70	− .37	.50	.25
Excessive exercise	EPSI-5	2.21	1.25	− .14	− .92	.49	.24
	EPSI-8	1.11	1.19	.81	− .33	.57	.33
	EPSI-22	1.31	1.16	.60	− .44	.59	.35
	EPSI-31	.82	1.06	1.27	1.0	.76	.58
	EPSI-41	1.37	1.22	.56	− .61	.73	.53
Negative attitudes toward obesity	EPSI-14	.93	1.18	1.11	.26	.46	.21
	EPSI-20	1.26	1.20	.68	− .44	.51	.26
	EPSI-26	1.68	1.49	.34	− 1.30	.64	.41
	EPSI-30	1.33	1.40	.68	− .85	.75	.57
	EPSI-38	.83	1.20	1.39	.89	.73	.54
Muscle building	EPSI-7	.19	.55	3.40	13.35	.64	.41
	EPSI-15	.28	.65	2.85	9.18	.80	.64
	EPSI-29	.27	.65	2.72	7.51	.79	.62
	EPSI-32	.75	.93	.98	.04	.43	.19
	EPSI-35	.44	.83	1.87	2.86	.46	.21

**Table 2:** Goodness of fit indices found for the 8-factor model of the eating pathology symptoms inventory and measurement invariance in different groups of Iranian adolescents

Confirmatory factor analysis													
	χ^2^	*p*	*df*	CFI	TLI	RMSEA	90% CI	SRMR					
CFA	2313.261	.001	917	.924	.918	.041	[.039–.043]	.056					

Measurement invariance													
Model	χ^2^	*p*	*df*	CFI	TLI	RMSEA	90% CI	SRMR	Model Comparison	ΔCFI	ΔTLI	ΔRMSEA	ΔSRMR
Gender													
Configural	3102.622	.001	1834	.930	.925	.039	[.037–.041]	.064	–	–	–	–	–
Metric	3270.691	.001	1871	.923	.918	.041	[.038–.043]	.065	Configural vs. Metric	.007	.007	.002	.001
Scalar	3339.564	.001	1908	.921	.918	.041	[.038–.043]	.064	Metric vs. Scalar	.002	.000	.000	.001
Strict	3434.854	.001	1953	.918	.917	.041	[.039–.043]	.066	Scalar vs. Strict	.003	.001	.000	.002
zBMI status, average weight vs. overweight (i.e., ≤ + 1SD and > + 1SD)													
Configural	2826.782	.001	1834	.942	.937	.034	[.032–.037]	.062	–	–	–	–	–
Metric	2961.453	.001	1871	.936	.932	.036	[.033–.038]	.063	Configural vs. Metric	.006	.005	.002	.001
Scalar	3108.001	.001	1908	.930	.927	.037	[.035–.040]	.062	Metric vs. Scalar	.006	.005	.001	.001
Strict	3242.692	.001	1953	.924	.923	.038	[.036–.040]	.065	Scalar vs. Strict	.006	.004	.001	.003
ED groups, adolescents with and without ED (i.e., EDE-Q global score, ≤ 2.5) ^a^													
Configural	2777.442	.001	1834	.936	.930	.034	[.031–.036]	.062	–	–	–	–	–
Metric	2969.576	.001	1871	.925	.921	.036	[.033–.038]	.062	Configural vs. Metric	.011	.009	.002	.000
Scalar	3120.425	.001	1908	.917	.914	.037	[.035–.040]	.062	Metric vs. Scalar	.008	.007	.001	.000
Strict	3327.594	.001	1953	.906	.905	.039	[.037–.042]	.065	Scalar vs. Strict	.011	.009	.002	.003
ED groups, adolescents with and without ED (i.e., EDE-Q global score, ≤ 2.5)^b^													
Configural	2642.888	.001	1828	.944	.940	.031	[.029–.034]	.060	–	–	–	–	–
Metric	2834.972	.001	1865	.934	.930	.034	[.031–.036]	.061	Configural vs. Metric	.010	.010	.003	.001
Scalar	2986.847	.001	1902	.926	.923	.035	[.033–.038]	.060	Metric vs. Scalar	.008	.007	.001	.001
Strict	3201.380	.001	1950	.915	.913	.038	[.035–.040]	.064	Scalar vs. Strict	.011	.010	.003	.004
Age groups, younger adolescents (10–15) vs. older adolescents (16–19)													
Configural	2969.487	.001	1834	.935	.930	.037	[.034–.039]	.064	–	–	–	–	–
Metric	3139.488	.001	1871	.928	.924	.039	[.036–.041]	.066	Configural vs. Metric	.007	.006	.002	.002
Scalar	3186.959	.001	1908	.927	.924	.038	[.036–.041]	.065	Metric vs. Scalar	.001	.000	.001	.001
Strict	3268.224	.001	1953	.925	.924	.038	[.036–.041]	.069	Scalar vs. Strict	.002	.000	.000	.004
Age groups, adolescents vs. college samples^1^													
Configural	4467.928	.001	1834	.952	.948	.041	[.040–.043]	.058	–	–	–	–	–
Metric	4952.038	.001	1871	.943	.940	.044	[.043–.046]	.061	Configural vs. Metric	.009	.008	.003	.003
Scalar	5051.758	.001	1908	.942	.940	.044	[.043–.046]	.060	Metric vs. Scalar	.001	.000	.000	.001
Strict	5306.770	.001	1953	.938	.937	.045	[.044–.047]	.065	Scalar vs. Strict	.004	.003	.001	.005

*CFI* Comparative fit index. *TLI* Tucker–Lewis index. *RMSEA* root mean square error of approximation. *SRMR* Standardised root mean square residual. ^1^Data were used from Sahlan et al.’s study [24]. ^a^Before freeing an error covariance among three items. ^b^After freeing an error covariance among three item

### Measurement invariance by gender

As shown in Table 2, change in fit indices (i.e., CFI, TLI, RMSEA, SRMR) between the configural and metric models met the criteria offered by Cheung and Rensvold [40] and Chen [41], providing support for metric invariance and suggesting that factor loadings were equivalent by gender. In addition, change in fit indices between the scalar and strict models provided evidence for strict invariance by gender, which supported **H2**.

### Measurement invariance by weight status

In support of **H2**, change in fit indices (i.e., CFI, TLI, RMSEA, SRMR) between the configural and metric models met the criteria offered by Cheung and Rensvold [40] and Chen [41], providing support for metric invariance and suggesting that factor loadings were equivalent by weight status. In addition, change in fit indices between the scalar and strict models provided evidence for strict invariance by weight status (see Table 2).

### Measurement invariance by clinical eating disorder group

As summarized in Table 2, change in fit indices (i.e., CFI, TLI, RMSEA, SRMR) between the configural and metric models and between the metric and scalar, and between scalar and strict models indicate that the scale is partially invariant among adolescents with and without clinically significant eating disorder symptoms. The model fit of metric invariance was not strong based on the ΔCFI (.011). As such, modification indices (MIs) were evaluated and applied to improve the fit of metric invariance. MIs suggested freeing an error covariance among 1–3 items; however, the model fit did not improve among one (ΔCFI = .011) and two (ΔCFI = .011) items. Thus, we examined freeing an error covariance among three items, which slightly improved model fit (ΔCFI = .10) (see Table 2).

### Measurement invariance by age (younger adolescents vs. older adolescents)

In support of **H2**, change in fit indices (i.e., CFI, TLI, RMSEA, SRMR) between the configural and metric models met the criteria [40, 41], providing support for metric invariance and suggesting that factor loadings were equivalent by age. In addition, change in fit indices between the scalar and strict models provided evidence for strict invariance by age (see Table 2).

### Measurement invariance by age (adolescents vs. college students)

In support of **H2**, change in fit indices (i.e., CFI, TLI, RMSEA, SRMR) between the configural and metric models met the criteria [40, 41], providing support for metric invariance and suggesting that factor loadings were equivalent by age. In addition, change in fit indices between the scalar and strict models provided support for strict invariance by age (see Table 2).

### Item reliability and internal consistency

Item reliability values range from .19 to .68 (see Table 1 for more information). In support of **H3**, the F-EPSI subscales demonstrated good internal consistency based on both Cronbach’s alphas and McDonald’s ω, with Cronbach’s alpha values ranging from .71 to .85 and classical McDonald’s ω ranging from .71 to .88 (see Table 4).

### Gender and weight status differences

Contrary to **H4**, adolescent boys endorsed significantly higher scores than girls on the Body Dissatisfaction and Binge Eating subscales. In line with **H4**, adolescent boys reported higher scores than girls on the Excessive Exercise and Muscle Building subscales. The other mean subscale differences were not significant across genders (see Table 3).

**Table 3:** Means (standard deviations), mean rank (sum of ranks), and t/z tests by groups for the F-EPSI subscales

	Boys (*n* = 134)	Girls (*n* = 779)	*t*/*z*	*p*	Effect size^a^
Subscales	*M* (SD)/Mean rank (Sum of ranks)	*M* (SD)/Mean rank (Sum of ranks)			
Body dissatisfaction	8.64 (6.17)	7.13 (6.34)	2.61	.010	.24
Binge eating	6.96 (4.93)	5.91 (5.37)	2.26	.025	.20
Cognitive restraint	4.21 (2.30)	4.35 (2.77)	.54	.59	.05
Purging	464.01 (62,177.50)	455.79 (355,063.50)	.43	.67	.14
Restricting	8.78 (4.05)	8.59 (4.49)	.50	.62	.04
Excessive exercise	9.14 (4.88)	6.43 (3.95)	7.08	.001	.61
Negative attitudes toward obesity	6.25 (4.48)	6.0 (4.66)	.59	.55	.05
Muscle building	578.29 (77,491.0)	436.14 (339,750.0)	5.98	.001	.20

	zBMI score < 1 (*n* = 774)	zBMI score > 1 (*n* = 139)			
Body dissatisfaction	6.47 (5.85)	12.24 (6.74)	10.44	.001	.91
Binge eating	5.61 (5.0)	8.59 (6.26)	6.22	.001	.53
Cognitive restraint	4.19 (2.69)	5.10 (2.65)	3.73	.001	.34
Purging	432.01 (334,372.0)	596.18 (82,869.0)	8.71	.001	.29
Restricting	8.64 (4.56)	8.47 (3.60)	.41	.68	.04
Excessive exercise	6.58 (4.24)	8.19 (3.76)	4.57	.001	.40
Negative attitudes toward obesity	5.85 (4.62)	7.03 (4.61)	2.77	.006	.26
Muscle building	463.54 (358,778.50)	420.59 (58,462.50)	1.58	.067	.05

	Without eating disorder (*n* = 765)	With eating disorder (*n* = 148)			
Body dissatisfaction	5.94 (5.36)	14.64 (6.02)	17.70	.001	1.53
Binge eating	5.34 (4.76)	9.78 (6.38)	9.76	.001	.79
Cognitive restraint	4.20 (2.71)	4.97 (2.56)	3.33	.001	.29
Purging	429.20 (328,339.50)	600.69 (88,901.50)	9.33	.001	.31
Restricting	8.43 (4.52)	9.60 (3.78)	2.96	.003	.28
Excessive exercise	6.61 (4.20)	7.97 (4.05)	3.74	.001	.33
Negative attitudes toward obesity	5.54 (4.42)	8.57 (4.90)	7.0	.001	.65
Muscle building	457.92 (350,305.50)	452.27 (66,935.50)	.62	.80	.02

	Younger adolescents (*n* = 566)	Older adolescents (*n* = 347)			
Body dissatisfaction	6.39 (6.06)	8.91 (6.48)	5.85	.001	.40
Binge eating	5.38 (4.97)	7.17 (5.67)	5.0	.001	.34
Cognitive restraint	4.43 (2.69)	4.15 (2.72)	1.53	.13	.10
Purging	445.15 (251,956.0)	476.33 (165,285.0)	2.24	.025	.07
Restricting	8.33 (4.53)	9.09 (4.22)	2.59	.010	.17
Excessive exercise	6.57 (3.97)	7.24 (4.54)	2.32	.020	.16
Negative attitudes toward obesity	6.08 (4.70)	5.96 (4.53)	.36	.72	.03
Muscle building	427.95 (242,219.0)	504.39 (175,022.0)	4.41	.001	.15

	Adolescents (*n* = 913)	College samples (*n* = 765)			
Body dissatisfaction	7.35 (6.34)	8.15 (6.17)	2.62	.009	.13
Binge eating	6.06 (5.31)	6.09 (5.09)	.10	.92	.01
Cognitive restraint	4.33 (2.70)	4.08 (2.54)	1.89	.058	.10
Purging	775.90 (708,392.50)	915.41 (700,288.50)	7.04	.001	.00
Restricting	8.62 (4.43)	7.85 (4.62)	3.46	.001	.17
Excessive exercise	6.83 (4.21)	6.04 (4.19)	3.84	.001	.19
Negative attitudes toward obesity	6.03 (4.63)	6.87 (4.78)	3.64	.001	.18
muscle building	819.25 (747,978.0)	863.66 (660,703.0)	1.94	.053	.00

^a^Effect size = We used this formula (z/√*N* [49]) to assess effect size of purging and muscle building subscales. We used Cohen’s *d* for the other subscales. Regarding purging and muscle building, we used *z* (Whitney U) tests, and for the other subscales, we used *t* (independent sample) tests. Regarding purging and muscle building, we used *Mean Rank (Sum of Ranks)*, and for the other subscales, we used *Means* (Standard Deviations)

In support of **H4**, adolescents with higher weight reported higher scores on most of the F-EPSI subscales. Restricting subscale scores were not different among adolescents with low-to-average weight vs. higher weight. In addition, Muscle Building subscale scores were not significantly different among adolescents with low-to-average weight vs. higher weight (see Table 3).

### Clinically significant eating disorder symptoms groups

As presented in Table 3, adolescents with clinically significant eating disorder symptoms endorsed higher scores on most of the F-EPSI subscales compared to adolescents without clinically significant eating disorder symptoms. Muscle Building subscale scores were not different among adolescents with and without clinically significant eating disorder symptoms.

### Age groups

In support of **H4**, older adolescents reported higher scores than younger adolescents on most of the F-EPSI subscales. Cognitive Restraint and Negative Attitudes toward Obesity subscales were not significantly different across younger adolescents and older adolescents (see Table 3).

In support of **H4**, younger adolescents reported higher scores than older adolescents on Restricting and Excessive Exercise subscales. The college sample endorsed higher scores than adolescents on most of the F-EPSI subscales (see Table 3). Binge eating, Cognitive Restraint, and Muscle Building subscales were not significantly different across adolescents and college samples.

### Convergent validity

In support of **H5**, most of the F-EPSI subscales demonstrated significant, positive associations with eating disorder symptoms (F-EDE-Q global score), binge eating (assessed through the F-EDE-Q), purging (assessed through the F-EDE-Q), and over-exercise (assessed through the F-EDE-Q) (see Table 4).

**Table 4:** Means, standard deviations, internal consistency, and correlations between F-EPSI Subscales, zBMI, eating disorder-related measures, and depression in adolescents

	Body dissatisfaction	Binge eating	Cognitive restraint	Purging	Restricting	Excessive exercise	Negative attitudes toward obesity	Muscle building
zBMI	.39***	.19***	.18***	.24***	− .09**	.19***	.07*	− .12***
Eating disorder symptoms	.62***	.35***	.17***	.36***	.14***	.26***	.26***	.12***
Binge eating	.25***	.44***	.00	.29***	.02	.08**	.19***	.14***
Purging	.10**	.10**	.03	.26***	.02	.09**	.08**	.15***
Over-exercise	.16***	.06	.13***	.30***	.01	.30***	.02	.14***
Depression	.50***	.36***	− .18***	.17***	.32***	.03	.16***	.27***
α	.85	.83	.71	.73	.72	.76	.76	.73
ω	.86	.83	.71	.74	.72	.73	.77	.73
*M*	7.35	6.06	4.33	.79	8.62	6.83	6.03	1.93
SD	6.34	5.31	2.70	1.92	4.43	4.21	4.63	2.53

*zBMI* Body Mass Index. *Eating disorder symptoms* Farsi Eating Disorder Examination Questionnaire (i.e., F-EDE-Q; combined restraint, eating concern, weight concern, and shape concern subscales). *Purging* Summed self-induced vomiting and laxative misuse items. *Depression* Subscale of the Revised Children Anxiety and Depression Scale (RCADS). Regarding the associations of purging and muscle building subscales with the variables, we used Spearman correlations, and regarding the other F-EPSI subscales, we used Pearson correlations. *α* Cronbach’s α. *ω* McDonald’s ω. ^*^p < .05, ^**^p < .01, ^***^p < .001

### Criterion validity

In support of **H5**, most of the F-EPSI subscales demonstrated significant, positive associations with zBMI and depression. The Restricting and Muscle Building subscales had significant, negative associations with zBMI. Cognitive Restraint subscale was significantly and negatively associated with depression (see Table 4).

## Discussion

The majority of our five hypotheses were supported by the F-EPSI adolescent data. In support of **H1**, the original 8-factor structure of the EPSI demonstrated acceptable fit to the data in the overall sample. The scale was invariant across genders, weight status, and age groups, supporting **H2**. Measurement invariance of the scale by clinically eating disorder group required freeing error covariances of one to three items in the eating disorder symptoms group. The items for which covariances were freed contained content related to body dissatisfaction, weight stigma, dietary restriction, and exercise, all of which are implicated in global eating pathology. The subscales demonstrated acceptable internal consistency and supported **H3**. Hypothesis four (**H4**) was *partially* supported; adolescent boys reported higher scores on the Excessive Exercise and Muscle Building subscales compared to adolescent girls, adolescents with higher weight reported higher scores on most of the F-EPSI subscales compared to adolescents of low-normal weight, and older adolescents reported higher scores than younger adolescents on most of the F-EPSI subscales. In support of **H4**, and most sub-scales were significantly correlated with zBMI, eating disorder symptoms, binge eating, purging, over-exercise, and depression, in support of **H5**. Thus, these findings establish the F-EPSI as a robust measure of multiple facets of eating pathology for use in Iranian adolescent populations.

The adolescent version of the F-EPSI overcomes multiple limitations inherent to the existing measures of eating pathology translated to Farsi. Previously, only the F-EDE-Q [1], F-EAT-26 [5], and F-ChEAT-20 [6] were available for administration among preadolescents and adolescents in Farsi. In validating the F-EPSI, the current study provides a psychometrically sound tool to assess the eating pathology symptoms more common in boys, such as muscularity and excessive exercise [7] and the behavioral features of eating pathology, including binge eating and purging. In line with our hypothesis and previous research among adolescents, boys endorsed higher muscularity and excessive exercise than girls [10, 16]. However, adolescent boys endorsed higher Body Dissatisfaction and Binge Eating scores than girls that were not in line with previous research among the US [10] and Iranian samples [1, 13]. One possible explanation could be the importance of muscularity which is common among Iranian adolescent boys (41.1%; 19). This urgency to attain muscularity may encourage adolescent boys to engage in binge eating to increase massive muscular bulk as it is applauded among Iranian men [45]. In addition, Iranian adolescent boys tend to consume dietary supplements; however, this consumption would increase boys’ weight that in turn may result in elevated body dissatisfaction in boys [21]. Notably, boys and girls reported comparable scores on restricting and cognitive restraint that was not in line with Richson et al.’ [10] study. It may be noted that boys tend to engage dieting behaviors to attain better shape and that is another reason boys endorsed higher body dissatisfaction. Furthermore, it is likely that body dissatisfaction and muscle building are inter-related. Thus, boys may endorse higher dissatisfaction with their body and that is why they may need to engage in muscle building to be satisfied with their bodies or vice versa. More precisely, recent studies [45, 46] reported that Western societal norms such as thin-ideal internalization, pressures for thinness, and social comparison were extended to Iranian boys. Relatedly, the effects of societal norms on eating pathology are comparable between Iranian and US cultures [47]. These societal norms sometimes may not be attainable and that, in turn, would result in higher eating pathology among adolescent boys. Taken together, the inclusion of this population in health promotion and prevention programs may, therefore, be increasingly important among adolescent boys. In addition, the F-EPSI assesses weight stigma and muscle building, both important components of eating pathology that are typically not quantified within existing eating pathology measures. The present findings extended previous studies in Western adolescents [16, 17, 22] and noted that weight stigma and muscle building related to other facets of eating pathology symptoms and depression in adolescents.

To our knowledge, this is only the second study to examine the factor structure and group level differences on EPSI sub-scale scores in an adolescent sample, and first to examine these in a non-clinical sample. Richson et al. [10] found the six-factor EPSI to be invariant across age as well as age-related differences in body dissatisfaction, restricting, cognitive restraint, purging, excessive exercise, and binge eating in a sample of recovery app users. Similarly, the current study demonstrated metric invariance by age, and that older adolescents scored higher than younger adolescents, in line with prior work by Mond and colleagues [11]. In accordance with a previous research [10] and our research hypothesis, adolescents endorsed higher restricting and excessive exercise than adults. However, contrary to Richson et al.’ [10] study and our research hypothesis, the college sub-sample scored higher than the adolescent sub-sample on most F-EPSI sub-scale scores than adolescents. Notably, both adolescents and college students endorsed similar scores on binge eating, cognitive restraint, and muscle building subscales. Overall, these findings indicate the importance of using the EPSI while assessing eating pathology symptoms across developmental periods in the future. Given that multiple facets of eating pathology are not similar in prevalence across developmental periods, clinicians need to consider it upon working on individuals with eating pathology in therapeutic settings in Iran. In addition, the current study extends Richson et al.’s [10] findings by establishing measurement invariance not only by developmentally, but also by gender, weight status, and clinically significant eating disorder symptomology status, making the F-EPSI a useful tool for both researchers and clinicians. In line with a previous study in Iran [14], adolescents with higher weight tended to report higher eating pathology symptoms.

The F-EPSI subscales were associated with zBMI and depression, supporting criterion validity of the scale. Consistent with the Iranian adult study [24], adolescents’ weight was negatively associated with restricting and muscle building. It can be noted that adolescents, irrespective of BMI, tend to use supplements to build muscularity and participate in eating-related restriction. The present study extended a previous studiy in Iran [14] and reported that F-EPSI and zBMI were inter-correlated, which may suggest that eating pathology is implicated more in Iranian adolescents’ zBMI, compared to Western societies [25]. In line with previous studies among patients with eating disorders [25] and college students [28], the F-EPSI subscales were related to depression. Interestingly, the associations between body dissatisfaction and depression were higher than previous studies among college students in China (*r* = .25; [28]) and general psychiatric patients in US (*r* = .29; [25]). It can be noted that adolescence is a period in which eating pathology could lead to higher depression or vice versa [48]. Thus, longitudinal studies need to examine the directionality of these associations. More precisely, cognitive restraint was negatively associated with depression which was not in line with a previous study among patients with eating disorders in US [25]. Thus, Iranian adolescents, regardless of depressive symptoms, may tend to engage in cognitive restraint to control body weight and shape. Future studies need to examine how other psychological symptoms such as anxiety symptoms affect cognitive restraint among adolescents.

## Strength and limits

This study is the first to empirically examine the eight-factor version of the F-EPSI in Iranian adolescents while assessing measurement invariance, reliability, and validity. The current study benefits from a large sample size, which provided the ability to examine the F-EPSI across gender and developmental periods. However, some limitations of the current study should be addressed in the future. First, the F-EPSI was not administered longitudinally, which prohibited the examination of both test–retest reliability and within-person change over time. However, Sahlan et al. [24] found that the F-EPSI scores are stable over time among adults in Iran. Second, the F-EPSI has only shown to be valid among younger adolescents, older adolescents, and emerging adults. It will be necessary to validate the F-EPSI in child (i.e., < 10), and adult (i.e., 28 >) populations, and patients with eating disorders, to ensure each conceptualizes eating disorder symptomology measured by the EPSI in the same way. Finally, we examined clinically significant eating disorder symptoms group using a cutoff of the EDE-Q global score established in Norway [37]. Thus, future studies need to validate a cutoff of the EDE-Q global score for use within an Iranian context. Relatedly, future studies need to include clinical samples to identify cutoffs through ROC curves for the F-EPSI subscales. These limitations notwithstanding, the current study provides a novel tool for assessing multiple facets of eating pathology among Iranian adolescent boys and girls.

## Conclusions

The F-EPSI was a psychometrically valid and reliable scale when applied to Iranian adolescents and could be useful in both research and therapeutic settings. This scale assesses eating pathology in both boys and girls and in our study it was invariant across genders, weight status, and eating disorder groups. In this way, the F-EPSI can be used across multiple developmental periods (e.g., younger and older adolescence and emerging adulthood). The findings suggest the F-EPSI as a robust measure of dietary pathology for use in Iranian adolescent populations.

## What is already known on this subject?

The majority of eating pathology measures focuses on girls’ eating pathology (e.g., thinness/dietary restraint) and do not reliably examine eating pathology more commonly associated with boys (e.g., muscularity/exercise). The EPSI is a relatively new assessment of eating pathology that addresses the limitations of previous eating pathology scales by capturing multiple dimensions of eating pathology cognitions and behaviors relevant to both boys and girls.

## What this study adds?

This study assessed the validation of the Farsi version of the EPSI (F-EPSI). The F-EPSI’s 8-factor structure was supported among adolescents. The F-EPSI was found to operate similarly across gender, age, weight status, age, and eating disorder groups, suggesting that scores on the scale can be compared across those groups. Furthermore, the scale demonstrated both reliability and validity.

## Data Availability

The data that support the findings of this study are available from the corresponding or first author upon reasonable request.

## Footnotes

1 As assessed by a global score of the Eating Disorder Examination Questionnaire (EDE-Q).

2 This program was used among Iranian schools.
